# Selecting an Appropriate Animal Model of Depression

**DOI:** 10.3390/ijms20194827

**Published:** 2019-09-28

**Authors:** Yuanzhen Hao, Huixiang Ge, Mengyun Sun, Yun Gao

**Affiliations:** 1 Joint Program of Nanchang University and Queen Mary University of London, Nanchang 330006, China; qmhorizon@126.com; 2 Department of Physiology, The Basic Medical College of Nanchang University, Nanchang 330006, China; huixiang.ge@qq.com (H.G.); 605697873@qq.com (M.S.)

**Keywords:** depression, animal model, behavior test

## Abstract

Depression has become one of the most severe psychiatric disorders and endangers the health of living beings all over the world. In order to explore the molecular mechanism that underlies depression, different kinds of animal models of depression are used in laboratory experiments. However, a credible and reasonable animal model that is capable of imitating the pathologic mechanism of depression in mankind has yet to be found, resulting in a barrier to further investigation of depression. Nevertheless, it is possible to explain the pathologic mechanism of depression to a great extent by a rational modeling method and behavioral testing. This review aims to provide a reference for researchers by comparing the advantages and disadvantages of some common animal depression models.

## 1. Introduction

Depression, as a common mental disorder that endangers both the physical and psychological health of the global population, is one of the most serious disorders and significantly contributes to the increasing suicide rate in the 21st century [1]. Depression leads to morbidity, and has an incidence that ranges from 13.3% to 17.1% in the United States [2]. A mild depression episode manifests as sadness, anhedonia, and a feeling of worthlessness, whereas the severe condition is classified by a recurring intention to commit suicide [3]. Clinically, with the unclear pathological mechanism and the inconsistent therapeutic effect, depression is considered to be a heterogeneous disease [3]. To date, animal modeling has played an essential role in exploring the underlying mechanism of depression. Studies have demonstrated that about 40–50% of the occurrences of depression are mediated by genes, indicating that it is necessary to select an appropriate animal model to locate the most susceptible gene [4]. By analyzing some of the core symptoms of depression that can be represented in a particular animal model, a specific drug or therapy can be developed and applied. Although it is currently unclear whether investigators will manage to identify the biological mechanism of depression based on the manifestations observed in animals, models of depression can still describe similar molecular alterations and pathogeneses to a great extent by utilizing appropriate criteria and methods [5]. Regarding behavioral presentation, it is widely accepted that a desirable animal model should fulfill three eligibility criteria: face validity, construct validity, and predictive validity [6]. In addition, a fourth criterion, called density validity, has been proposed to evaluate the reliability of genetic variation [7]. These four criteria determine the credibility of an animal model of depression. Based on observed validities and practical experience, this review summarizes some commonly used animal models of depression, and compares the advantages and disadvantages of different modeling methods. It aims to provide a more feasible modeling reference for investigators and suggest selection protocols for animal models of depression.

## 2. Validity Provisions

In general, it is accepted that three criteria can be used to assess the reliability of an animal model of depression: the phenomenological or morphological appearances (face validity), a similar etiology (construct validity), and therapeutic similarities (predictive validity) [8,9]. Through these criteria, the modeling efficiency can be determined. However, some clinical symptoms of depression, such as self-denial, dysthymia, and suicide predisposition, are hard to precisely detect [10]. In addition to the propositions of the gene-environment theory and mismatch theory, it is necessary to highlight that a model’s accuracy may be reduced by diversity in the genomes of experimental animals [11]. Moreover, these three conventional validities have some defects, such as low practicality, measurement difficulties, and gene-environment incompatibility [12]. For the purpose of overcoming these limitations, population validity has been proposed as a fourth criterion. This validity criterion, which emphasizes the interaction between the genetic type and developmental circumstances, requires that the different depressive symptoms in distinctive animal models should match the respective human clinical conditions [7]. These four validities offer a complete management strategy to investigators, and play an essential role in evaluating animal models of depression.

## 3. The Molecular Mechanism in Animals that Mimic Human Depression

There are several interacting molecular alterations within depressive animals. Dysregulation of some neurotransmitters can evoke a neurobiological disruption that can be observed among patients with depressive disorders [13]. These neurotransmitters in the nervous system include: (1) monoamines (the overuse of reserpine can deplete monoamines and induce depression in patients and animals [3]); (2) gamma amino butyric acid (GABA), which is an inhibitory amino acid neurotransmitter whose reduction has been observed in the ventral hippocampus and frontal cortex of animals with depression [14,15]; (3) glutamate, which is an excitatory amino acid neurotransmitter (the glutamate level in the brains of animals with depression was found to have increased within 24 h and decreased over the next four weeks, and a reduced concentration of synaptic vesicle protein vesicular glutamate transporter-1 (VGLUT-1) in the CA1 region of the hippocampus was detected [16,17]); (4) corticotrophin-releasing hormone (CRH) and arginine vasopressin (AVP), which are two neuropeptides that participate in the hypothalamic-pituitary-adrenal axis and contribute to depression in the hypothalamus [18]; and (5) brain-derived neurotrophic factor (BDNF) (a low level of BDNF is a crucial parameter in the animal depression modeling process [19]).

Furthermore, an excess of oxidative stress and aberrations in the inflammatory pathway are two important components of depression [20]. Depression induces a decrease in the concentration of plasma, vitamin E, zinc, and coenzyme Q10, which participate in the antioxidant process. A low antioxidant capacity reduces protection against reactive oxygen species (ROS), causing an oxidative stress status, impairing the metabolism of fatty acids, proteins, and DNA, and producing an excess of xanthine oxidase, malondialdehyde (MDA), and 8-hydroxy-2-deoxyguanosine [21]. Oxidative stress may also stimulate autoepitopes to escape immunosurveillance and lead to an autoimmune response that attacks the neurons in the brain, causing neurodegenerative disorders. It has been demonstrated that some antidepressants, such as zinc and N-acetyl-cysteine, can reverse depression in some animals by consistently elevating antioxidant levels and normalizing the disruption in the brain [20].

Many more molecules than those mentioned above have been reported to be involved in depression. However, the exact molecular mechanism remains unclear. Therefore, in order to explore the molecular mechanism that underlies depression, different kinds of animal models of depression are used in laboratory experiments.

## 4. Depression Modeling Approaches

### 4.1. Reserpine-Induced Model

Depression contributes to alterations in various biochemical indexes, such as a deficiency of monoamine metabolites, an increased concentration of inflammatory factors, and a decreased level of BDNF in serum [22]. Therefore, it is feasible to establish animal models of depression by altering the concentration of particular physical molecules. Reserpine, as a vesicle reuptake inhibitor, can arrest the reuptake process of the presynaptic membrane. When blocked outside the vesicle, the transmitters are degraded by monoamine oxidase. Furthermore, the depletion of catecholamines (such as norepinephrine, epinephrine, and dopamine) and serotonin (5-HT) may lead to morphological changes in animals, including ptosis, hypothermia, and motorial inhibition [3]. Additionally, the phenotypes of this model can be readily observed, including ptosis, hypomotility, and diarrhoea [23]. It has been shown that the reserpine-induced progressive model of depression has high face validity for anxiolytic compounds, which has promising implications for the treatment of anxiety-induced depression [24]. Recently, it was reported that reserpine-induced depression is associated with heightened, fluoxetine-sensitive, ethanol consumption in female, but not in male, adolescent rats. Thus, this model is currently used to explore the relationship between depression in adolescent females and alcohol consumption [25].

Depression in this model can be reversed by the intake of antidepressants in advance, indicating that reserpine induction is a potential method for modeling depression that is able to evaluate the effect of antidepressants [11]. Various drugs have recently been investigated to ameliorate depressive behaviors that are specifically established by the reserpine-induced method. For instance, a traditional Korean medicine called Gyejibokryeong-hwan (GBH) has been reported to potentially regulate immune/endocrine dysfunction in mice to mitigate depression via activating the BDNF-cAMP-response element binding protein (CREB) pathway [26]. α-(phenylselanyl) acetophenone (PSAP), which is an organic selenium compound with antioxidant characteristics, can reverse the pain-mediated depression induced by reserpine via inhibiting type-A monoamine oxidase (MAO-A) [27]. Cocaine and amphetamines, as two blockers of monoamine reuptake, may produce agitation rather than a mood-relief effect in patients with severe depression [28].

The reserpine-induced model has the following advantages: (1) it is less time-consuming; (2) it is able to distinguish between the pharmacological effects of general antidepressants and those of other psychoactive drugs; (3) it has medium predictive validity; and (4) regarding ethical considerations, it is only slightly painful for animal models [29].

However, the reserpine-induced model also has the following obvious disadvantages: (1) the animals have a high mortality rate; and (2) the model has an inconsistent pathological process with individual depression, which may have an impact on the construct validity of the animal models. In general, the mechanism that underlies depression among humans is associated with long-term stress or a mental burden. Therefore, it is possible that results obtained using the reserpine-induced model have reduced reliability [6].

### 4.2. Learned Helplessness Model

Learned helplessness is a depression model in which animals manifest a low intention to escape in an environment of uncontrollable and unpredictable injury stimulation. The catecholamine level is the main measurement in this model, which has high predictive validity [30]. The most commonly used method for building a learned helplessness model is tail shock [31], which consists of a triadic design and a foot shock in shuttle boxes [32,33]. In this model, it should be noted that rats provide a better presentation when incorporating a lever press, and mice are more suitable for the shuttle box paradigm [33,34]. Several behaviors, including rapid eye movement sleep [35], diminished sexual behavior [36], and an increased concentration of corticosterone [37], can be observed in this model and assist researchers with their investigation of depression in animals.

Regarding drug administration, several novel compounds that have a potential therapeutic effect on this model have been discovered. For example, Curculigoside (CUR), which is extracted from the traditional Chinese medicine Curculigo orchioides Gaertn (Xianmao in Chinese), was reported to facilitate fear memory and ameliorate depression in mice via stimulating the Akt-mTOR signaling pathway and increasing BDNF expression [38].

The advantages of this model are that: (1) the model animals manifest depression-like symptoms that basically cover the onset of depression in humans, and most acute symptoms can be reversed by antidepressants within 3–5 days [39]; (2) the correlation between cognitive impairment and behavior caused by autonomic nervous system dysfunction in rats is obvious, which provides a model for investigating nerve circuits in the course of depression onset; and (3) it is easy to study the impact of genetic variations in different species on depression-like symptoms [33,40].

The disadvantages are as follows: (1) this model has a short depression duration [10]; (2) the subjective criteria have a great impact on the behavioral results; and (3) different species of model animals have different stimulated responses [41,42].

The learned helplessness model has a selective and specific effect on commonly used antidepressants. So, it has relatively high face, predictive, and population validity. It is suitable for investigating the etiology of depression and use in the antidepressant manufacturing process.

### 4.3. Chronic Mild Stress Model

In general, a single repeated stimulation triggers an adaptation response under normal conditions. However, the chronic mild stress (CMS) model can overcome this adaptation and produce long-term and effective depression in an animal group [43]. This method for long-term inescapable stimulation comprises a series of trials, such as electric shock, ice walking, fixation, day and night reversal, tail clipping, and water or food deprivation, that are randomly performed every day. After 3 weeks, behavioral tests, including open-field and sucrose preference tests, are carried out. The preliminarily established model can produce a range of positive results, such as a reduced cycle number in the open-field test and a decreased preference for sugar water [40]. With the development of this model, the intensity of stimulation has been limited to standardizing the various factors that can have an impact on the results [40,44]. The most obvious feature that a model animal manifests is anhedonia, which is a basic symptom of depression patients, and this characteristic is assessed by measuring the amount of consumed sugar solution and comparing it with water consumption [45]. Once this model induces behavioral alterations in rats, it will last for approximately three months. Most antidepressants can reverse the effects of reduced sucrose consumption [39,46]. Currently, the CMS model is widely used and studied to investigate the underlying mechanism, etiology, and emerging animal lines of depression [47,48]. For example, Wistar Kyoto (WKY) rats were shown to be predisposed to a depression-like phenotype in response to chronic mild stress, with the underlying mechanism that WKY rats exhibit more obvious changes in neurotransmitter and neurotrophin levels than Sprague Dawley (SD) control rats [49]. Regarding sexual predisposition, it is difficult to diametrically define whether a male or a female animal model has a higher likelihood of becoming depressed under chronic mild stress conditions. Different theories result in different consequences, which may become a contentious point in the depression modelling field [50].

A user survey indicated that the success of this model depends on the interaction between the individual variety of susceptibility to stress and the intensity of the applied microstimulators. It also showed that the quality of a CMS model can be elevated by training laboratory operators to use sophisticated techniques [51].

Regarding emerging therapeutic treatments, subacute and chronic ketamine treatment was demonstrated to have antidepressant, anxiolytic, and precognitive effects by means of the CMS model with great reliability in 2017 [52]. Several drugs, including ginsenoside Rg2, ethanolic extract of *Capsicum pubescens* fruits (EEFCP), icariin, and lurasidone, have been shown to exert a similar antidepressive action in the past five years [53,54,55,56].

This model has the following advantages: (1) reliable predictive validity, as antidepressants previously used to treat patients have a great therapeutic effect on CMS animals. This model can be used to select the most optimal antidepressant for further research on the pathological mechanism of depression [57]; (2) obvious face validity, as CMS not only simulates the clinical symptoms of most depression cases in humans, but also emphasizes anhedonia as a crucial and measurable feature; and (3) great construct validity, as CMS shows a close causal effect between risk factors of depression and treatment methods [58].

The shortcomings of the CMS model are quite prominent: (1) the multiple high-frequency protocols of the CMS model require a larger number of operators and a huge amount of experimental space, and result in relatively low efficiency; (2) based on the high-density stimulation, the mortality of model animals is higher than that in other methods; and (3) different experimental environments and evaluative criteria may lead to errors in results [46,58].

### 4.4. Social Defeat Stress Model

Social defeat stress is a long-term and repeated stimulation. Considering the circumstances of actual human life, most depression cases are triggered by the high-pressure social environment rather than primary neural circuit damage [59]. To obtain great construct validity, the social defeat stress paradigm simulates the pathogenesis of depression at a social level. Therefore, the social defeat stress model has been gradually established over time [60,61]. With a frequently performed stimulation, model rodents manifest a stress response to produce long-term behavioral and psychosocial changes [62,63], such as a reduced frequency of communication with groups [62] and a reduction in sexual behavior [64]. It is worth mentioning that the gender of rodents in this model is male, indicating that researchers are required to artificially create low-aggression intruders to fight with resident rodents [65]. In consequence, despite the high incidence of females who suffer from depression in reality, the social defeat stress model only focuses on male animals. Therefore, Takahashi et al. [66] established the repeated social defeat stress model by chemogenetically activating the ventrolateral subdivision of the ventromedial hypothalamus of mice to induce aggressive behaviors among female animal models. Williams et al. [67] have recently demonstrated that administration of the kappa opioid receptor antagonist could block the occurrence of anhedonia (in both males and females) and social avoidance reactions (in females only). These findings indicate that some short-acting antidepressants have different therapeutic effects within different genders, and depression is more likely to be established in female rats within the social defeat stress model.

Some researchers have found that rats living in a group had a lower probability of exhibiting long-term depression-like behaviors than rats living alone [64,68]. Previously, some studies found that naive rats suffered from depression after 5 weeks of cohabitation with depressed rats in the same cage [69].

Regarding therapeutics, several novel drugs have been discovered in recent years. For example, (R)-ketamine, as the most popular drug in this model, is widely understood to play an antidepressant role by activating the extracellular-signaling-regulated kinase (ERK) pathway [70]. Within nearly three years of investigations, the therapeutic effect of (R)-ketamine was compared with rapastinel, ethosuximide, and lanicemine, which highlights how heated the field of the drug’s future applications is [71,72,73].

The advantages of the social defeat stress model are as follows: (1) excellent face and construct validity, as it is possible to simulate most cases of depression in humans; (2) the model demonstrates that social interaction can slightly alleviate the degree of depression [64]; (3) chronic antidepressants partially reverse the symptoms of depression in this model [74]; and (4) a shorter modeling time than the average modeling time of depression (20 days) and a simple modeling process [75].

The main disadvantages are: (1) the symptoms in this model can be confused with symptoms of anxiety, which may mislead investigators [65]; and (2) this model mainly simulates the underlying mechanism of depression in male patients in a social environment and ignores gender-based differences [76].

### 4.5. Other Models

In addition to the above four models, several novel modeling methods, such as the lesion model and the chronic oral exposure to corticosterone (CORT) model, have been proposed in the last decade. The lesion model involves surgical or chemical removal of the olfactory bulb to produce depression-like behavioral abnormalities in rodents. However, the exact mechanism by which a bulbectomy induces depression symptoms remains unclear. This model is now rarely used because it has low predictive validity for antidepressant tests and a high morbidity rate [39]. In the CORT model, animals accept oral corticosterone, which is a stress-related adrenal hormone, in regular intervals to induce persistent depressive behaviors, such as anhedonia and low motivation [77]. These models may facilitate the investigation of complicated stress-related depression symptoms in the future.

With the advance of research on genetic variations, mutant approaches provide an opportunity to discover potential risk factors of depression. It has been demonstrated that various lines of mice can contribute to different results on depressive predisposition. For example, α_2A_ adrenergic receptor knockout mice and high-expression cAMP response element-binding protein mice can manifest depression symptoms more easily after treatment in a stressful environment [78,79]. Female Flinders Sensitive Line (FSL) rats manifest an obvious depressive-like phenotype when undergoing treatment with selective serotonin reuptake inhibitors (SSRIs) [80]. Probiotics were found to reduce the risk-taking behavior in FSL rats with depression [81]. The Wistar Kyoto More Immobile (WMI) genetic model describes the independent pathological mechanisms for the environmental and genetic etiologies of depression [82].

## 5. Behavioral Tests

### 5.1. Forced Swimming Test (FST)

The forced swimming test (FST) rat model was originally established by Porsolt and his colleagues in 1977, and was subsequently used to perform behavioral tests in mice [83,84]. In the FST, animals are subjected to inescapable stress by tail suspension and eventually manifest an immobile posture. The majority of antidepressants can effectively reverse this state of immobility. The FST has been used to test the antidepressant potential of drugs for decades [85].

Compared with the anxious behavior of normal animals, this immobility was first seen as a form of desperate behavior. Subsequent evidence showed that the immobile state of FST rodents actually reflected an extinction-like inhibitory learning behavior that was caused by the inescapable/unavoidable feature of the FST. The immobility produced by the FST can be regarded as a switch that promotes cognitive functioning for an animal’s adaption and survival [86,87,88]. However, the notion that desperate behaviour can represent depression has been queried, and recently some researchers have expressed doubt as to whether the FST is a good depression model [89].

The controversy is not limited to the original design of the FST. The reactive baseline of rodents interacting with antidepressants is inconsistent among different species. For example, the FST yields negative results for DBA/2 mice, such that they cannot be evaluated by performing the FST method. The ddY mice can swim for longer than other species of mice [84]. In addition, the primary dyskinesia of animals may contribute to errors in results. It is necessary to apply the open-field test (OFT) in advance to eliminate the false depression group that is generated by mobility deprivation [83].

The serum level of interleukins IL-6 and IL-15 can also be regarded as an indicator of a depressive state in animals, and provides a novel pathway for depression therapy [90]. Over the last three years, the FST has been used as the main test for evaluating both the modelling and drug-therapeutic efficiency. However, the main problem with the studied drugs, including fluoxetine, tipepidine, and tramadol, was the low statistical power for clinical applications, which may not provide a direction for meta-analyses [91,92,93].

The advantages of the FST are that: (1) it is a cheap and fast method that applies very large numbers of compounds [84]; (2) it is highly automated, providing an effective means to test a model group; and (3) it is a broad-spectrum method for testing the efficiency of antidepressants with strong predictive validity.

The shortcomings of the FST are that: (1) it has a high mortality rate; (2) it cannot be used to evaluate the etiologic mechanism of depression in both animals and in humans; (3) there are too many environmental interruption factors to accurately measure the immobility time of the forced swimming test (IMFST)

Although the FST and OFT (see below) cannot be used to conduct etiologic investigations, their high efficiency has made them popular behavioral test methods for selecting antidepressants [94].

### 5.2. Tail Suspension Test (TST)

The tail suspension test (TST) was proposed by Steru and colleagues in 1985, has a similar depression induction mechanism to the FST, and also manifests an immobile posture [95]. Similarly, the TST was found to not be suitable for detecting a “desperate mood” in animals. Instead, it is suitable for detecting a transition from active to passive behavior due to unbearable environmental stress in a copy or survival manner [88].

Antidepressants that reverse this immobility and stimulate escape behavior can be seen as therapeutic medications. However, the dose-response curve of these two behavioral tests is different in the case of an intervention with antidepressants [96]. In addition, the baseline of immobility duration is different among different species of rodents. For example, the immobility time of Swiss mice in the TST is seven minutes shorter than that in the FST, while this phenomenon does not occur in C57BL/6 mice [96]. It can be considered as an indirect proof of the necessity of approving the construct validity. The immobility time can be reduced by typical or atypical antidepressants, including SSRIs and monoamine oxidase (MAO) inhibitors. For instance, nimodipine can perform an antidepressant-like action by forming liposomes to disrupt the MAO-B receptor [97].

The advantages of the TST are that: (1) it can simultaneously measure the efficiency of antidepressants in a range of model animals; (2) it has low expenditure and man power requirements; (3) it can function as a supplementary test to the FST (under some specific conditions, such as hypothermia, the TST can replace the FST to quantify animal behaviors); and (4) it can be applied to both mice and rats. Compared with the low accuracy of the FST, the TST, which presents only a mild risk to animals, can be considered to be a gentle stimulation method that is not life-threatening.

The disadvantages of the TST are as follows: (1) it is an incomplete protocol in the sense that it is a supplementary method that relies on other tests; and (2) the pharmacodynamic effect of antidepressants in the TST occurs too quickly to predict the appropriate treatment course for depressive patients.

### 5.3. Open-Field Test (OFT)

The emotional state of animals can be manifested by their initial behavior when they are trapped in a totally isolated and unfamiliar environment. The open-field test (OFT) imitates unsafe surroundings, evaluates animals’ autonomous behavior, and reveals how tense the animals are [43,98]. Generally, animals fear a novel environment and continually move in the surroundings of a black box and seldom in its central area. However, their natural characteristic of making exploratory attempts will encourage them to explore the central area, which allows investigators to observe the depressive state of rats. By using a three-dimensional (3D) imaging sensor to track the movement of animals, investigators can easily observe instances of reduced activity of depressed animals, which run for less time [98]. In a previous method for the FST, each rat that undergoes the OFT should be wiped down to remove any odor that may attract other rats. The commonly used antidepressants have therapeutic effects on rats in the OFT.

The advantages of the OFT are as follows: (1) its semi-automatic operation (similar to the FST) makes it more effective and convenient than other tests; (2) it results in less harm to animals; (3) it has a great predictive effect for therapeutic similarity; (4) it is great for measuring fear and despair; and (5) it has a dual function, as it can be used to test anxiety-like animal models.

The disadvantages of the OFT are that: (1) it is time-consuming; (2) it has low construct validity for the investigation of etiology; and (3) it is sensitive to alterations in the environment.

### 5.4. Sucrose Preference Test (SPT)

The sucrose preference test is commonly used to evaluate anhedonia in animals, which refers to a reduced capacity to experience happiness [57]. A decrease in the sucrose consumption ratio of an experimental animal is indicative of a successful depression model with anhedonia. It is widely accepted that the SPT is the most appropriate behavioral test for the chronic mild stress model. In psychopathology, anhedonia and pressure sensitivity are the two main internal phenotypes that indicate an extremely severe depressive state. It also represents a fatigue or loss of energy condition under depressive stress, which can be reversed by antidepressants. Regarding the mechanism for measuring the ratio of consumption of water and sucrose solution, a reduced intake of sucrose indicates that an animal is in a depressed state.

The advantages of this test are: (1) it is the most convenient test for anhedonia in depressed animals; (2) it results in less harm to animals; and (3) it is suitable for use in studies on the comorbidity of depression and pain.

The disadvantages of this test are as follows: (1) it is a time-consuming test that takes three days to prepare; and (2) it is too rough to accurately indicate the depressive state of animals.

### 5.5. Other Behavioral Tests

The novelty-suppressed feeding (NSF) test is based on hyponeophagia and provides a sensitive model for screening the efficiency of antidepressants [99]. The NSF uses quantity of food intake and latency period for searching for food as its essential parameters. This test exhibits some advantages as an animal model for exploring the neurobiological aspects of antidepressant actions [99]. For example, the NSF produces a contradictory situation for animals the motivation to eat, and the fear of venturing into the center of an open area with an intense bright light. These two competing conditions have great construct validity for studying an anxious mood during a treatment course of antidepressants [100]. The latency of the NSF test is causally associated with the function of hippocampus neurogenesis, which provides an opportunity for investigators to study the interaction between a neural circuit and an antidepressant effect [101,102]. However, the NSF test cannot be simultaneously performed with other behavioral tests, which is its greatest shortcoming.

The elevated plus maze (EPM) test focuses on the anxiolytic effect on animals and is similar in some degree to the OFT [103]. As depression is often accompanied by anxiety, the EPM test may be an effective method for assessing changes in anxiety behavior when combined with the OFT test [104,105]. Cognitive deficits have also been associated with major depressive disorder. Thus, a cognitive test, such as the spontaneous alternation test, can also be performed on a depressed animal [106].

## 6. Potential Treatments forAnimal Models of Depression

Several antidepressants have a potential therapeutic effect on animal models of depression. Ketamine is a proven drug in clinical applications; administration of a subanesthetic dose evokes a rapid and effective antidepressant effect. It has been proven in the CMS and social defeat stress models that ketamine has a less delayed onset of antidepressant effects (hours or days) compared with other antidepressants (typically weeks) [107]. Ketamine can modify synaptic plasticity and continuously stimulate excitatory synapses. However, abuse and the dissociative property of ketamine restrict its widespread use [108]. In the reserpine-induced model, ketamine and rapastinel have been demonstrated to have an antidepressant-like effect under treatment-resistant conditions. S-ketamine and rapastinel are promising therapeutics that target the glutamatergic pathway in patients with depression. However, more follow-up data are needed before conclusions can be drawn on their potential effect [109]. A combination of agomelatine/tianeptine and adenosine A_2A_ receptor antagonist (DMPX) represents a potential treatment for the learned helplessness model in mice [110]. Imipramine is a tricyclic antidepressant that can disrupt DMPX in a pharmacodynamic manner and provide a therapeutic effect. It has been evaluated in a forced swimming test and a tail suspension test [110,111]. Fluoxetine is an SSRI that could ameliorate depression by blocking astrocyte activation and repairing oligodendrocyte dysfunction. It is usually used in models, such as the CMS model, where depression is concomitant with anxiety or neuropathic pain [112].

## 7. Combining Animal Models of Depression

Once experience with different modeling methods had been obtained, the direction of investigation moved from macroscopic operations to the combination of macroscopic and microscopic operations. However, considering that depression is a multifactorially induced neurological disorder, it is insufficient to focus on the results of modeling alone. Based on the above mentioned articles, we suggest that depression modeling should employ specific methods in each of an animal’s growth stages. With the foundation of an accurate genotype, each modeling procedure needs to be evaluated by the four validity criteria. Moreover, in the absence of strict recommendations, interventions may be carried out in an early phase to elevate the incidence of depression in an animal’s adult phase. The unpredictable accuracy of the selected criteria is the main problem with modeling depressed animals. Therefore, every test should be confirmed by detectable molecular alterations within the animal’s body, and a real-time investigation of the animal’s brain pathology is essential in order to ensure that the modeling result is accurate. In particular tests, such as the FST and the TST, coping and learning behaviors were often roughly equated with depression and sometimes referred to as “depression-like” features. This loose terminology simultaneously oversimplifies the complexity of the neurology and misrepresents the applications and limitations of various behavioral tests [113]. Therefore, a more accurate terminology should be used to promote investigations into animal models of depression.

## 8. Conclusions

Different modeling methods involve different experimental procedures, which produce different selectivities upon antidepressants with both advantages and disadvantages (See Table 1). Therefore, it is difficult to predict the therapeutic effect using only a single animal model. The pharmacologic model has low reliability but high efficiency, which enables it to be used to select antidepressants. So, it is a convenient method for a laboratory that does not have enough time to model, and it is suitable for some concomitant disorder modeling processes. The learned helplessness, chronic mild stress, and social defeat stress models have high specificity, which indicates that these animal models can manifest symptoms that are similar to those of depression in humans. These three methods may benefit investigators who study the etiology of, or perform drug research in, depression. The incidence of false positive results can be raised by using a single method, which are easily impacted by subjective preferences. So, we recommend these three methods to laboratories that have sufficient time and funds. A number of behavioral tests play an indirect role in modeling depressed animals. The forced swimming test and the open-field test play a common role in depression modeling. However, they have low specificity. The tail suspension test is easy to use to investigate the characteristics of depression due to the obvious morphologic alterations that it produces. The sucrose preference test can be used to determine whether the anhedonia symptom is present in a depressed animal. A successful modeling process should be comprised of different methods in order to elevate the incidence of depression in animals.

## Figures and Tables

**Table 1:** Advantages and disadvantages of animal models of depression.

Depression Modeling Approaches	Advantages	Disadvantages
Reserpine-induced model	1. Medium predictive validity 2. Less time-consuming 3. Distinguishes different pharmacological effects	1. High mortality rate 2. Low construct validity
Learned helplessness model	1. High construct and face validity 2. Covers almost all depression symptoms 3. Imitates neural circuit alterations of depression 4. Benefits genetic investigations	1. Short depression duration 2. Easily affected by subjective impacts 3. Different species effects
Chronic mild stress model	1. Great face, construct, and predictive validity 2. Benefits the selection of optimal antidepressants 3. Measures anhedonia 4. Helps to find risk factors of depression	1. Can waste resources and labor 2. High mortality rate 3. Easily affected by environmental impacts
Social defeat stress model	1. Great face, construct, and predictive validity 2. Manifests social interaction 3. Shorter modeling duration	1. Can be confused with anxiety 2. Cannot model female animals
Behavioral tests		
Forced swimming test (FST)	1. Cheap and fast 2. Highly automated 3. Broad spectrum for antidepressants 4. High predictive validity	1. High mortality rate 2. Cannot evaluate the etiologic mechanism 3. The immobility time of the forced swimming test (IMFST) measure is insufficiently precise
Tail suspension test (TST)	1. An effective test 2. Low expenditure and man power requirements 3. A supplement to the FST 4. Applies to both mice and rats	1. Incomplete persuasion protocol 2. Excessive pharmacodynamic effect of antidepressants
Open-field test (OFT)	1. Effective and convenient 2. Less harm to animals 3. Great predictive effect for therapeutic similarity 4. Can detect fear and despair	1. More time-consuming 2. Insufficiently accurate 3. Sensitive to alterations in the environment
Sucrose preference test (SPT)	1. Can manifest anhedonia 2. Less harm to animals 3. Great for investigating other comorbidities	1. A time-consuming test to prepare 2. Low accuracy
